# Online reputation of agri-food companies and determining factors: an empirical investigation

**DOI:** 10.1007/s11846-023-00639-8

**Published:** 2023-03-15

**Authors:** Domingo Fernández-Uclés, Adoración Mozas-Moral, Enrique Bernal-Jurado, Raquel Puentes-Poyatos

**Affiliations:** 1 Department of Business Organization, Marketing and Sociology, University of Jaén, Building D3, office 006, Campus Las Lagunillas, s/n, 23071 Jaén, Spain; 2 Department of Business Organization, Marketing and Sociology, University of Jaén, Building D3, office 146, Campus Las Lagunillas, s/n, 23071 Jaén, Spain; 3 Department of Economics, University of Jaén, Building D3, office 266, Campus Las Lagunillas, s/n, 23071 Jaén, Spain; 4 Department of Business Organization, Marketing and Sociology, University of Jaén, Building D3, office 132, Campus Las Lagunillas, s/n, 23071 Jaén, Spain

**Keywords:** Online reputation, Olive oil, Website, Social networks, fsQCA, 39B82, Q13, P13, G14

## Abstract

In an increasingly technology-oriented society, companies should ensure not only that they have an Internet presence but also that they are conveying the right image. According to the resource-based view, online reputation is a key intangible asset for successful technological business change. The aim of this research is to analyze the online reputation of companies in the agri-food sector, identifying the factors that have an impact on it. For this purpose, fuzzy-set qualitative comparative analysis is used. The results show that online reputation is conditioned by legal form and attributes associated with the company website. Such attributes include website quality, the presence of information associated with corporate social responsibility, the use of a secure connection, and the sale of organic products. The results provide strategic guidelines for public and private decision makers to exploit the full potential of ICTs.

## Introduction

Olives and olive oil are a fundamental part of Spain’s image, both inside and outside its borders. According to the International Olive Council (2022a, 2022b), in the 2020/2021 season, Spain produced 67.72% of European olive oil and 44.82% of all olive oil worldwide. Andalusia in the south of Spain is the main olive oil producing region in the world. In the 2020/2021 season, it produced 80.11% of Spanish olive oil, 54.29% of European olive oil, and 36.99% at the global level. Therefore, Spain and, more specifically, Andalusia are the main actors in the global olive oil market. In Spain, the sector employs more than 350,000 farmers and 15,000 industrial workers (milling, bottling, extracting, and refining). It offers more than 32 million working days per season, according to data from the Spanish Ministry of Agriculture, Fisheries, and Food (MAPA 2020).^1^

As in the agri-food industry as a whole, the olive oil sector is burdened with a serious commercial problem, resulting in low prices. The high sale of bulk product as inputs for other industries rather than reaching the end market, the excessive atomization of this supply compared to the high concentration of the distribution sector, and the lack of professionalization and training of its human capital are some of its main problems (Mozas 2019). Several studies suggest that this lack of market orientation can negatively affect innovation in the firm (Ho et al. 2018).

In response to the above problems, the use of information and communication technologies (ICTs), particularly the Internet, can offer solutions to these problems (Fernández et al. 2020). Accordingly, in a context of low prices, the use of the Internet of Things (IoT) can increase yields and the profitability of the food supply chain (Kamilaris et al. 2016). In commerce, the presence of a website as a landing page for Internet users, the existence of a virtual store as an online sales channel, and the use of online social networks as a communication channel and relational marketing strategy can increase competitiveness within this sector and, therefore, enhance the efficiency of resource use (Wei et al. 2013). The adoption of ICTs in business is fundamental for the development and growth of organizations, as well as for achieving competitive advantages (Kaplan and Haenlein 2010). ICTs are leading to digitization of traditional company functions. They are also creating new opportunities for business and digital entrepreneurship (Kraus et al. 2019; Nambisan et al. 2019). Some studies have linked ICT adoption with developing new sustainable mechanisms and green product innovation performance (Muñoz-Pascual et al. 2021). They have also highlighted links with the possibility of creating innovation networks (Lyytinen et al. 2016). In addition, technological innovation reduces transaction costs, improving the efficiency of business actions along the value chain (Evans and Wurster 1997). The economic, social, and environmental sustainability promoted by the United Nations through the 2030 Agenda (United Nations 2015) is shaping the actions of many companies in their quest to improve their competitiveness. The challenge facing the business community is to identify these opportunities (Khanin et al. 2022) and turn them into innovative business models (Aström et al. 2022) that will positively affect their reputation (Rubio-Andrés et al. 2022).

In order to be successful in leveraging the potential of the Internet in commercial operations, a company’s ability to achieve a positive online reputation is crucial. Online reputation can be understood as the result of a company’s activities in a virtual environment, with the subsequent interactions and reactions of stakeholders (Kanika 2016). A positive online reputation attracts customers and causes them to change their attitudes toward the company, increasing sales and improving both the company’s image in the market (Horster and Gottschalk 2012) and the economic value associated with a brand or sector (Casado-Molina et al. 2020). Hence, online reputation, or e-reputation, can be considered one of the greatest intangible assets of companies (Deephouse 2000).

Numerous studies have examined online reputation (Rantanen et al. 2020), most of which have focused on a single platform, mainly in the service sector (Veh et al. 2019). This study addresses online reputation in a more holistic way, covering consumer reviews on Google and major virtual social networking sites. Its focus on the olive oil industry makes it a pioneering study that can be extrapolated to other products in the agri-food sector. The specific objective of this paper is to measure the level of online reputation of olive oil companies and to identify which factors make them most attractive to their current or potential customers. Specifically, it analyzes to what extent the quality of websites, the communication of corporate social responsibility (CSR), security in navigation (SSL), the legal form of the company, and its organic offering contribute to enhancing its online reputation for customers.

In order to address its aims, the paper is structured as follows. After this introduction, the scientific literature related to the research objective is reviewed. Then, the methodology is presented, and the analysis model is proposed, in this case based on fuzzy-set qualitative comparative analysis (fsQCA). Finally, the results and discussion section is presented, followed by the corresponding conclusions.

## Literature review and model construction

Since the 1980s and early 1990s, the focus of competitive advantage analysis has shifted from industry and competitor analysis to the analysis of internal aspects of the firm, specifically the exploitation of unique internal resources and capabilities. The resource-based view explains why organizations competing in the same competitive environment, which are therefore subject to the same factors, achieve different competitive positions. According to the resource-based view, the specific characteristics and attributes of a company and the use and combination of these attributes can contribute to its competitive position (Abimbola and Kocak 2007; Ferreira et al. 2019). From this perspective, reputation is an intangible resource that can generate competitive advantage for an organization, given that it is a valuable, rare asset that is difficult to imitate and replace (Barney 2007). According to Casado et al. (2020), corporate reputation has two main components: emotional (affective) and rational (cognitive). Both of these components are defined by the relationship between experiences, emotions, and attitudes. Thus, consumers’ brand, product, or company experiences can explain both components, eliciting emotions and, in turn, generating a certain attitude (Conway and Briner 2002).

According to Lemon and Verhoef (2016), an organization’s reputation can be investigated through consumer experience. Many authors have investigated the variables that explain consumers’ reviews of their experiences with a brand through digital media (Fombrun and Gardberg 2000; Ramos et al. 2019). These variables can be grouped into six dimensions (Fombrun and Gardberg 2000; Ponzi et al. 2011): product, ethics, management, work environment, social responsibility, and organizational profitability. This article is aimed at identifying organizational characteristics that are related to these dimensions, such as CSR communication, the commitment to organic products, the use of social economy organizational models, and so forth. It is argued that actions in response to the growing concerns of citizens in areas such as quality, social engagement, and environmental conservation contribute to a company’s online reputation (Martín de Castro 2021).

Reputation is understood as a network of perceptions about a company’s ability to meet the expectations of all stakeholders (Fombrun 1996). There is no generally accepted definition of reputation, although scholars agree that it is the sum of the perceptions of each stakeholder (Fombrun et al. 2015; Dowling 2016). Accordingly, the reputation of a company built through digital channels or its website is defined as the image that stakeholders have of the company, based on the content of the website and other virtual platforms (Marchiori and Contoni 2011). This reputation is called online reputation, or e-reputation, and can be understood as an extension of traditional reputation (Dutot and Castellano 2015).

Like traditional reputation, online reputation is a value that is difficult to control and measure (Marchiori and Contoni 2011; Rantanen et al. 2020), to the extent that the same brand can have different reputations depending on the stakeholder group that evaluates or assesses it (Orozco and Ferré 2017). Thus, despite the existence of a multitude of indices at the business level (World’s Most Admired Companies, Reputation Quotient, RepTrak, and MERCO) or academic level (Dowling 2016; Gupta et al. 2017), there is no consensus on the attributes or dimensions that make up the reputation of a company.

According to Dutot and Castellano (2015), interactivity and trust, credibility of data sources, and security and confidentiality of transactions are specific attributes of online reputation. The ethical behavior of companies, protection of the environment, and support for good causes are also cited as key factors of reputation (Reputation Institute 2021). Other factors affecting reputation are the quality of products and services provided by the company and its innovative potential, as it reflects the ability to meet customer needs (Zraková et al. 2019).

It follows that the attractiveness of companies to their customers depends increasingly on the reputation conveyed through social networks and the company website (Luca 2011), which has a positive impact on financial performance (Ramos et al. 2021). Consequently, as a result of the development of ICTs, no company or organization should ignore the digital dimension of reputation and the paramount importance of its management within the company’s corporate communication, marketing, and customer service strategy (Alzamora et al. 2021; Khan et al. 2019). Thus, to leverage the value of digital technologies, companies must design, develop, and implement digital business model innovation (Trischler and Li 2022).

### Website quality and security

In the online environment, the website is the medium through which many potential customers will have their first contact with the company. Therefore, it will be their first reference when forming an image of the company (Garaus and Wolfsteiner 2022; Chen and Macredie 2005). The ability of the website to influence users’ impressions of the company positively may determine their final decision on whether to make a purchase (Vand Der Heijden et al. 2004). This decision is conditioned by the potential of the information provided to compensate for the absence of personal contact between agents and generate sufficient trust between them (Mckinnev et al. 2002).

Despite a lack of unanimity when it comes to determining the characteristics that a website must have in order to be positively valued, there is a certain consensus in that the presence of a high level of information, interactivity, and services is positively related to user satisfaction (Heinze and Hu 2006). Many studies have tried to identify the most appropriate metrics to assess the effectiveness of a website as a business information system (Heinze and Hu 2006; Yang et al. 2005). Most of them have been carried out under the technology acceptance model (Lee et al. 2003).

Several authors (Yang et al. 2005; McKinney and Zahedi 2002) have argued that the quality of information and the quality of the system are the two main determinants of users’ perceived usefulness and ease of use of an information system. The quality of information is determined by the usefulness of its content and its ability to enable users’ choices. These choices are in turn influenced by variables such as timeliness, relevance, and accuracy (Delone and Mclean 1992). The quality of the system refers to its potential or capacity to retrieve and distribute information. It can be measured through the presence on the website of features such as interactivity, privacy/security, or useful links (Mckinney and Zahedi 2002).

A critical factor in inducing consumers to make online purchases is whether they have sufficient assurance of confidentiality in the transfer and processing of information. Security is recognized as an attribute in the use, perception, and reputation of companies in the virtual environment (Davidavičienė et al. 2019; Ključnikov et al. 2019). Evidence is the growing importance of blockchain technology to improve cybersecurity in the exchange of information (Aslam et al. 2021).

Proposition 1. Website quality contributes to a company’s online reputation.

Proposition 2. The presence of a secure website connection (SSL) contributes to a company’s online reputation.

### Sustainability and social responsibility

Society’s concern about environmental degradation and a healthy lifestyle has led to a growing demand for information on sustainability and CSR. Research in marketing shows that consumers prefer products from companies that invest in environmental protection actions and behave well toward society (Nadanyiova et al. 2020). Thus, consumers attribute higher quality to products from socially responsible companies (Mercade-Mele et al. 2017; Cui et al. 2018).

Reflecting this idea, in recent years, consumer appreciation of CSR actions and the Sustainable Development Goals (SDGs) has been gaining importance (Randle et al. 2019). CSR has been shown to be increasingly relevant in the formation of corporate reputation (Almeida and Coelho 2019). CSR acts as a means for reputational risk management and as a tool for generating customer and employee loyalty and attracting socially responsible investors (Freeman 2006). Hence, online communication of CSR plays a key role in interacting with and providing information to stakeholders. Media choices are essential, as indicated by Mercade-Mele et al. (2017). The medium influences the perception of CSR, in addition to enabling more rational decision making by stakeholders (Wanous 1992). For example, Christensen et al. (2017) indicated that external certifications or standards are one of the tools that organizations can use to communicate their CSR practices and benefit from the advantage of offering maximum assurance by being certified by third parties.

In parallel, growing social concern for healthy eating and greater awareness of environmental issues have given organic agriculture an increasingly greater presence in the shopping baskets of consumers. Specifically, in Spain, over the period 2015 to 2020, spending on organic products grew by 67.42% (MAPA 2020). Certain agricultural products, such as organic products, are experiential because of their ability to evoke sensations and convey experiences (Schmitt 1999). Such products are particularly suitable for online marketing (Stricker et al. 2007) because their purchase and consumption require an intensive exchange of information (Giampetri et al. 2018). This intensive exchange of information refers not only to tangible aspects of the product but also to others such as symbols, tradition, culture, tourism, and gastronomy, which can significantly enhance the value perceived by consumers (Canavari et al. 2002). In this context, the Internet is one of the main sources used to search for food information (Kuttschreuter et al. 2014). Social networks provide information and confidence to consumers to compensate for the lack of knowledge associated with organic food (Evelyn et al. 2015).

Proposition 3. Commitment to CSR contributes to a company’s online reputation.

Proposition 4. The presence of organic products in a company’s online offering contributes to the company’s online reputation.

### Social economy

Cooperatives are built on certain principles established by the International Cooperative Alliance (ICA).^2^ These principles include inter-cooperation, which, according to the ICA, serves to strengthen the cooperative movement by working together and creating local, national, regional, and international structures (ICA 1995). It has a positive impact on the propensity of individuals to create agricultural startups (Arafat et al. 2020). The advantage of integration as a pillar of cooperatives is intended to achieve the same outcome as concentration, namely a greater organizational size. A cooperative is itself a form of integration, given that it is an agreement between many individual entrepreneurs who share their means of production to reap the benefits of collaboration and thus earn greater profits (Mozas 2019). The rate of cooperatives in the Andalusian olive oil sector (i.e., the percentage of oil produced by cooperatives) is almost 70%. However, the number of cooperatives accounts for 50% of organizations in the sector (MAPA 2022). The importance of cooperatives in this sector is unquestionable.

Cooperatives are stable enterprises unconditionally linked to the rural environment and farmers, who therefore exercise leadership in the local economy and in the connection of people to the land. Cooperatives thus contribute to the balance and management of a region, making them true agents of rural development (Monzón and Chaves 2012). Some authors consider that cooperative societies, by their very nature, act under cooperative principles and values that make them exponents of socially responsible enterprises and, therefore, crucial for sustainable development (Mozas et al. 2020). Thus, the contribution of social economy (SE) entities and cooperatives to the SDGs has not gone unnoticed by either the United Nations or the organizations representing the SE and other relevant institutions (Mozas 2019).

Consequently, Martínez and Eid (2017) linked the higher reputation of cooperatives than that of other enterprises to two dimensions: one related to more ethical behavior (responsibility) and another to a greater concern for society (citizenship). Thus, in SE entities, intangibles are the most important forms of value they possess (Audretsch et al. 2022). Within these intangible forms of value, reputational value stands out. Transparency and mediatization are the best exposure techniques to generate reputation (Rosado 2016). One example that supports the above statement is that cooperatives directly or indirectly provide a great deal of employment in the rural world because they protect it and play the role of economic stabilizer (Cancelo et al. 2022).

Similarly, in times of crisis such as the current situation of COVID-19, cooperatives are more resilient, projecting an image of stability and solidity (Cancelo et al. 2022). Commitment to society and ethical behavior have a decisive impact on the reputation of these entities (Díaz and Marcuello 2010). Also, although Spanish olive cooperatives have shown a certain delay in the adoption of ICTs with respect to other legal forms, they have greater popularity on social media than companies with other legal forms. Thus, their actions on these platforms are more efficient (Fernández et al. 2016). It has also been shown that the cooperative legal form in other sectors positively affects popularity on social platforms (Jimena et al. 2021).

Proposition 5. The cooperative legal form contributes to a company’s online reputation.

## Data and method

### Data collection

The study population was olive oil organizations in Andalusia, Spain, with their own website. This region is the largest producer of olive oil in the world. With respect to the national total, Andalusia accounted for 80.11% of Spanish olive oil production in the 2020/2021 season. Therefore, the importance of Andalusia in this area is undeniable.

The list of olive oil processing organizations was obtained from the Food Information and Control Agency (AICA),^3^ an autonomous agency affiliated with the Spanish Ministry of Agriculture, Fisheries, and Food. The first step was to identify the websites of these organizations on leading search engines. Of the 826 olive oil organizations in the list, 466 had a website. Notably, 53.86% of these organizations were cooperatives. The data were extracted from the Internet, both directly from the organization’s website and with the support of web tools specialized in the analysis of these platforms. The data were obtained between September and October 2021.

### Method

Qualitative comparative analysis (QCA) provides a powerful tool for the analysis of causal complexity, based on Boolean algebra. Its verbal, conceptual, and mathematical language gives it the advantages of both qualitative and quantitative analysis, offering a hybrid approach (Schneider and Wagemann 2012). QCA allows systematic analysis of a set of causal factors that combine to result in a given outcome. Different combinations of causal factors can lead to the occurrence of the same outcome (equifinality). They can also have opposite effects depending on their combination with other factors (Schneider and Wagemann 2012). The validity and usefulness of the technique has been demonstrated in the literature, especially in social science studies (Rihoux et al. 2013; Pappas and Woodside 2021).

Its strength with respect to conventional quantitative methods is that it establishes relationships between subsets of conditions to explain patterns in the data in the form of relationships of necessity and sufficiency (Ragin 1987). QCA is valid on its own. The assumption is that asymmetry, equifinality, and causal complexity may exist. This assumption mitigates some of the limitations of multiple regression (Ragin et al. 2006). QCA evaluates cases as configurations of causes and conditions rather than considering each independent variable. QCA thus offers greater explanatory power and a richer view of relationships of interest (Gligor and Bozkurt 2020). Another of its strengths is that it is ideal for research designs with small to medium-sized samples (Kumar et al. 2022). Of the three strands of QCA (csQCA, fsQCA, and mvQCA), fsQCA is used in this study. Its characteristics make it more suited to the proposed objective.

### Outcome and condition measurement

#### Outcome

The outcome was a construct that captured the online reputation of olive oil organizations. There is no consensus on the dimensions of online reputation (Veh et al. 2019), so the indications of Ahmed and Rodriguez (2020) were followed. They considered both quantitative ratings, where satisfaction variables were measured with a measurement scale, and qualitative ratings, based on the opinions of users. From the customer perspective, recent research has analyzed online reputation through data mining in social networks (Samaggia et al. 2019; Borrero and Zabalo 2021) and digital tools such as Social Mention (Gémar and Jiménez 2015; Dutot et al. 2016). This study is built on the research of Štefko and Pollak (2016) and Dorčák (2017), adapted to the unique features of the focal companies.

Following these arguments, the outcome of online reputation (OR) was defined as follows: OR = (GR + SS + FO)/3. Each element is described later. All elements were previously transformed so that they had the same weight in the construction of the OR outcome. The purpose of this construct was to capture weighted information and consumer reviews about the organization and its products on major Internet platforms.

- *Scores from Google reviews and Google star ratings (GR)* Users can rate organizations on this platform on a scale of 1 to 5 stars, where 5 is the highest score. The resulting score is calculated from the ratings of all users. Google aims to ensure that the overall score accurately reflects the quality of the company. The overall score received by the company, together with the number of reviews or ratings received, was considered to compile this outcome.
- *Sentiment extracted from Social Searcher (SS)* This web tool is one of the most prestigious for analyzing the reputation of a brand or company on the Internet. It enables the search for content on social networks in real time and provides analytical data on user ratings on the main social networks: Twitter, Google+, Facebook, YouTube, Instagram, Tumblr, Reddit, Flickr, Dailymotion, and Vimeo. To compile this outcome, the difference between the total number of positive and negative comments by users about each company was used.
- *Followers on social networks (FO)* The number of users who follow the companies on three of the main social networks used at the business level (Facebook, Twitter, and Instagram) was employed. This attribute measures the company’s overall online influence on consumers.

#### Conditions

**Fig. 1:**
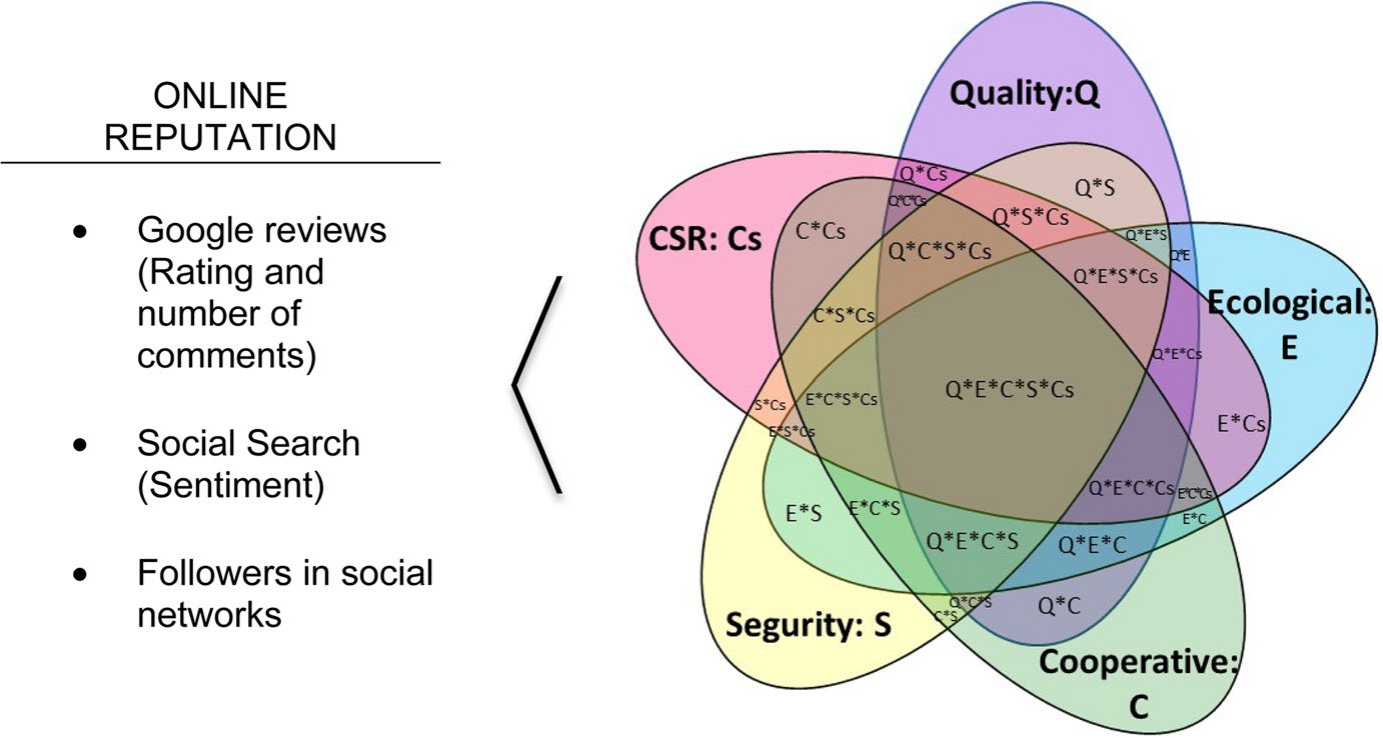
Conceptual configural modeling of antecedent conditions for the importance of online reputation. Source: Authors

Based on the literature review, five causal conditions were used: the presence or absence of an organic offering on the company website; the existence or absence of a secure domain, providing consumers with confidence in the website; the legal form of the organization (cooperative or not); the level of quality of the organization’s website (assessed using different items); and the presence of CSR indicators on the website. Figure 1 illustrates the structural model for this study.

Specifically, the website quality condition was constructed based on the indicators established in the Extended Model of Internet Commerce Adoption (eMICA). In total, 35 items that determine the degree of development of a website were reviewed, considering three dimensions: promotion phase (information), provision or interactivity phase (dynamic information), and processing phase, related to the degree and quality of transactions. Researchers such as Cristobal et al. (2020) and Fernandez et al. (2020) have compiled the items used to measure the level of website quality.

The CSR condition refers to the company’s commitment to CSR, as measured by the certifications or standards it communicates on the website. Given that there is no single CSR certification, the national and international certifications or standards that exist for the dimensions covered (social, economic, and environmental) are an informative indicator of the CSR commitments of organizations (Rasche 2010). Consequently, the condition was measured through six indicators, following the work of Mozas et al. (2020). Each indicator took the value 0 or 1 depending on whether it had any of the accrediting certifications. Table 1 shows, in greater detail, the certifications used.

**Table 1:** Items used for website analysis of CSR

Dimension	Indicators and certifications
Social	Food quality and safety ISO 9001/Denomination of Origin/BRC/IFS/ISO 22000/GLOBAL GAP/ISO 22005 Traceability
	Labor and occupational safety ISO 45001/OHSAS 18001/SA 8000/EFR Model (Family Responsibility)/SEDEX—SMETA Supply Chain Responsibility
Environmental	Environmental quality of activity ISO 14001/Zero waste/FSC—PEFC/ISO 50001 Energy efficiency management/Integrated Production Andalusia/ISO 14064 Carbon footprint of organizations
	Product environmental quality GlobalEPD—Environmental Product Declaration/Product Carbon Footprint/Water Footprint/CAAE Organic Agriculture/Organic Agriculture Certificate—EU
Economic	Economic transparency; financial reporting of activity
	Responsible management. Codes of conduct or ethics/SR10/EFQM Model/Sustainability report/SGE 21/ISO 26000

Source: Compiled by the authors

Table 2 shows all conditions and the outcome considered in this research.

**Table 2:** Conditions and outcomes used in fsQCA

	Description	Type
*Outcome*		
Online reputation	Sentiment toward the organization among Internet users, measured through the ratings and comments of consumers	Continuous*
*Condition*		
Quality	Development of the organization’s website (score obtained through eMICA)	Continuous*
CSR	A company’s commitment to CSR, measured by the certifications or standards shown on the website	Categorical**
Security	Presence of a security protocol on the organization’s website (SLL, HTTPS)	Dichotomous***
Cooperative	Cooperative legal form of the organization	Dichotomous***
Organic	Presence of organic offering on company website	Dichotomous***

Source: Authors

*Continuous conditions/outcome were calibrated using fsQCA 3.1 software

**Categorical condition with five levels of a company’s commitment to CSR. Calibrated as per Rihoux and Ragin (2009)

***Dichotomous condition. A value of 1 indicates presence of condition, 0 its absence

### Descriptive statistics

As an initial overview of the situation of the organizations analyzed in this study, Table 3 shows basic statistics on the outcome and conditions used in this research.

**Table 3:** Descriptive statistics of the analyzed organizations

Condition	Details
Quality	6.86% of organizations have a website of maximum quality
CSR	46.35% of organizations communicate about CSR
Security	54.07% of organizations have a website with a secure connection
Organic	16.95% of organizations sell organic products
Cooperative	53.86% of organizations are cooperatives

	Valid N	Mean	Standard deviation	5th percentile (fully out)	Median (cross-over point)	95th percentile 95th (fully in)
Online reputation	466	.47	.19	.17	.46	.80
Quality	466	.47	.31	.05	.50	.95
CSR	466	.23	.25	.00	.25	.75
Security	466	.54	.50	.00	1.00	1.00
Organic	466	.17	.38	.00	.00	1.00
Cooperative	466	.54	.50	.00	1.00	1.00

Source: Authors

The above descriptive values show that, on average, the analyzed organizations operate under the legal form of a cooperative and are not committed to offering organic products to help them generate greater added value. In relation to the use of a website, a basic tool for operating in the digital environment, there are clear deficiencies. Only a small number of the websites of the organizations analyzed in this study have high-quality attributes. Just over half offer a website with an SSL secure connection. This attribute directly affects the use and the positive perception that consumers form about the company. Among the informative content of these websites, most do not provide information on their CSR, thereby missing an opportunity for consumers to attribute extra quality to the products they offer.

## Results and discussion

Following the recommendations in the literature, all causal conditions and the outcome were calibrated so that their measures in fuzzy sets had values ranging from 0 to 1. Subsequently, a necessity analysis of the outcome for the different causal conditions was performed to verify that none explained the outcome by itself. In none of the cases was the consistency equal to or higher than the recommended limit of 0.9. No coverage was too low, with all registering values above 0.5 (Ragin 2006).

The fsQCA technique is not symmetrical. It is useful to study which combinations (or configurations) of factors lead to a low level of online reputation because, for a certain configuration that explains the outcome, the opposite does not always explain its negation. Thus, two possible models are presented, depending on the consistency cut-off point for the negative result.

The first model has adequate coverage but unacceptable consistency. The second model has low coverage and optimal consistency. However, in the first model, the ~ Security* ~ Quality and ~ Cooperative* ~ Quality* ~ CSR configurations have raw coverages above 30% and consistencies of 80–85%. Thus, with a total coverage of 60.2% and a consistency of 82.03%, both configurations lead to a low reputation. Hence, in the analyzed sector, there is still ample room for improvement. Low levels of reputation are associated with a low overall website quality and the rest of the associated parameters.

**Fig. 2:**
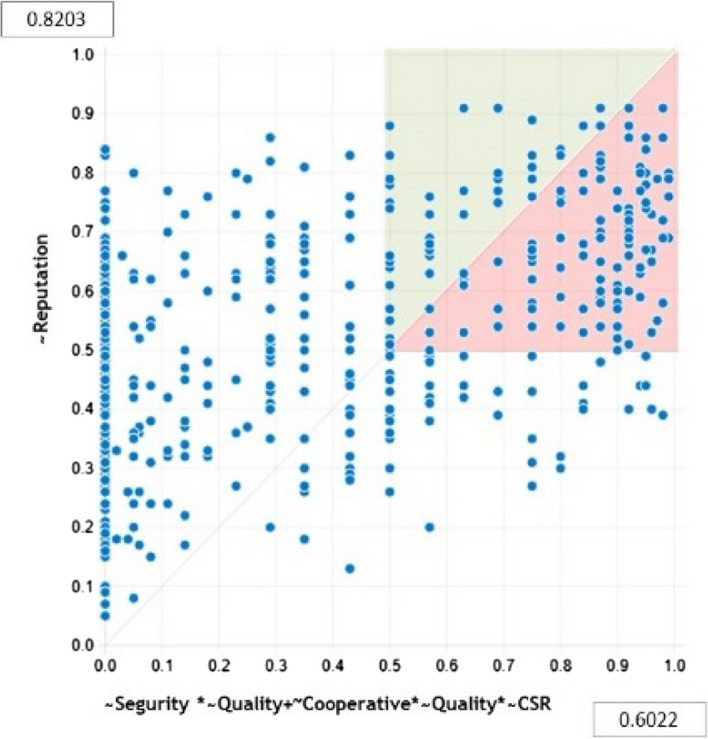
Representation of the model for opposite cases. Source: Authors

To represent the consistency and coverage of a solution, scatter plots of the solution versus the outcome can be used. Companies below the diagonal are inconsistent with the outcome, and those above are consistent. However, within each group there are degrees of relevance depending on whether the combination membership score is less than or greater than 0.5 (upper right quadrant): more relevant is inconsistent in the lower shaded triangle (Yi >  = 0.5, Xi > Yi), and more relevant is consistent in the upper shaded triangle (Xi >  = 0.5, Xi <  = Yi). Figure 2 depicts the model results for the analysis of opposite cases.

To analyze sufficiency, the next step is to run the fuzzy-set algorithm and generate the truth table (Iannacci and Kraus 2022). Once the configurations with low frequency (remainders) had been eliminated, a consistency threshold of 0.8 was established, identifying the natural breakpoints and considering that the minimum recommended value is 0.75 (Rihoux and Ragin 2009). Subsequently, the solutions of the proposed model were obtained. The results are shown in Table 4.

**Table 4:** Analysis of sufficiency

Configurations	1	2	3	4	5	6
Quality	●	●	●		●	●
CSR	●			•		
Security		●	●	⊗	$\bigotimes$	
Organic			●		$\bigotimes$	●
Cooperative		●		•	$\bigotimes$	●
Raw coverage	0.3715	0.2538	0.1170	0.0941	0.0939	0.0708
Unique coverage	0.0831	0.1174	0.0277	0.0209	0.0547	0.0105
Consistency	0.9284	0.8060	0.7753	0.8099	0.7679	0.8134
Model coverage	0.6123					
Model consistency	0.8013					

Source: Authors

The results show the different combinations that lead to a higher reputation of the company on the Internet, considering the proposed conditions. Table 4 includes the set-theoretic consistency values for each configuration, as well as the overall model solution. The overall solution is above the recommended threshold of 0.80 (Pappas et al. 2021). The overall model resulting from this analysis reflects a total coverage of 0.6123. Thus, 61.23% of olive oil companies with a high online reputation are explained by this set of causal combinations or configurations.

The first of these configurations, with a gross coverage of 0.3715, establishes that the combination of a high-quality website, linked to CSR communication, explains 37.15% of cases of a high online reputation. Hence, it shows that the relationships between these conditions are those that explain a high online reputation. The corresponding consistency shows that 92% of cases present such a result. The second configuration suggests that the combination of a high-quality website, a secure connection, and cooperative status explains 25.38% of cases with a high reputation. In this case, the consistency indicates that 81% of cases present such a result.

The above results indicate that a company’s online reputation is directly related to the quality of information and interaction on the website (Chiu et al. 2005; Ye et al. 2019). It is also related to the security of transactions on the website (Davidavičienė et al. 2019; Ključnikov et al. 2019), the communication of CSR through the website (Miller et al. 2018; Almeida and Coelho 2019), and whether the oil is organic (Canavari et al. 2002; Evelyn et al. 2015). In view of these results, all propositions raised in this research may be accepted.

## Conclusions

The present study extends research into online reputation in two ways. First, it proposes a measurement indicator based on user sentiment and perceptions about digital platforms. Second, it reveals a configuration of conditions that positively enhance online reputation, which offers an intangible asset that provides a source of competitive advantage. This section presents the principal conclusions of the study. Emphasis is placed on the theoretical and practice contributions of the research. The study’s limitations and future lines of research are also highlighted.

### Theoretical contributions

The development of resources and capabilities to create a competitive advantage has become the primary goal of strategy design. Under the resource-based view, online reputation can be considered a key intangible asset to create competitive advantage for organizations through differentiation from competitors (Abimbola and Kocak 2007). Numerous studies have attempted to measure online reputation (Sylvaine and Dutot 2013; Rantanen et al. 2020). However, most are based on partial information (Dutot et al. 2016). They overlook the fact that stakeholders develop opinions and points of view based on the information provided through a range of technology-based sources such as websites, social media, blogs, and forums (Zraková et al. 2019). Likewise, most studies have primarily focused on the service sector (Veh et al. 2019), overlooking the study of other sectors that deal in tangible products, such as the agri-food sector. This study enriches the existing scientific literature by presenting a comprehensive indicator of online reputation based on the opinions of users on Google and leading social media platforms. It is applicable to any other agri-food sector product. Moreover, the study identified a combination of technological, organizational, and environmental factors that positively influence the creation of this business resource and that can therefore provide a source of competitive advantage.

### Practical implications

The results have major practical implications for agri-food sector firms, as well as society as a whole. The results offer insight into the actions that decision makers should take to harness the potential of ICTs.

The study shows that combinations of attributes such as cooperative status, online security in business-user relations, corporate social responsibility (CSR), and the sale of organic products are positively related to the online reputation of organizations. These attributes are closely linked to the Sustainable Development Goals (SDGs) of the 2030 Agenda. For instance, the United Nations (Inter-Agency Task Force on Social and Solidarity Economy 2014, 2015) highlighted the importance of the social solidarity economy, indicating that it could play a key role in meeting the 2030 Agenda goals. Cooperatives are one of the key families of the social economy. In SDG 17, ICTs are cited as a crucial tool to achieve the SDGs. The global adoption of ICTs is advocated as a way of supporting the creation of innovation, science, and technology capabilities with the end goal of promoting development. The underlying cybersecurity technology can generate consumer trust. Therefore, firms should focus on cybersecurity to improve their competitive position. Another key area is CSR. The World Business Council on Sustainable Development (WBCSD 2002) defined CSR as “the continuing commitment by business to behave ethically and contribute to economic development while improving the quality of life of the workforce and their families as well as of the local community and society at large.” According to Lizcano (2020), the perfect alignment between the goals of CSR and the SDGs is such that meeting CSR goals entails directly or indirectly meeting the SDGs, and vice versa. Therefore, CSR and the SDGs are fully interconnected, even though different methods are used in their application. This rationale should show companies that the search for alignment between the SDGs and CSR will give them greater recognition with citizens, who will therefore be more inclined to purchase sustainable products, as reported in some studies (PwC 2015). Finally, organic products link a company’s orientation with environmental protection, specifically SDGs 7, 13, 14, and 15. Therefore, this study shows that a company’s orientation toward meeting the goals of the 2030 Agenda is viewed positively by social agents online. It can thus give companies a better online reputation, providing a business resource that, according to some authors, has a direct relationship with business performance (Batrancea et al. 2022). Therefore, the study gives firms reasons for engaging in the attainment of the SDGs.

Managers can learn other lessons from this study. First, the study highlights the importance of an online presence and the opportunities provided by this presence given the ongoing growth in e-commerce operations at the national and international levels. Only 56.41% of firms in the focal sector have a website and are thus able to harness its potential to develop the key internal resource of positive online reputation. In addition, the study also shows the importance of having qualified professionals to administer a high-quality, secure website and furnish it with content that is relevant for users. Finally, managers should be aware that a commitment to CSR or organic production is not enough. In addition, they must also communicate this commitment through online channels so that it translates into a strong company reputation.

As a final practical implication, the results offer cooperatives an additional incentive to continue to work under this business structure. Their link to sustainability and the local community, their commitment to the development of rural areas, and their focus on the activity of a large number of producers, which also results in greater competitive capabilities, mean that these firms are better rated by users and, therefore, achieve a better online reputation. Therefore, the results provide arguments for both public and private decision makers to encourage the creation and development of social economy enterprises such as cooperatives and other business partnerships.

### Limitations and future research

It is also important to highlight the main limitations of this study. First, this study focused on olive oil sector firms. However, it is expected that the contributions of the study can be extended to many areas of the agri-food sector. This sector generally faces similar underlying sales and marketing problems. Another limitation is that the study focused on the Spanish national market. Even though Spain is a leading producer of olive oil, it would be of interest to compare the situation in other oil producing countries.

As a closing remark, this study provides a novel contribution to the agri-food sector literature by offering insight into the under-researched issue of measuring online reputation and finding combinations of conditions that are positively related to it. Given the importance and relevance of these topics, proposals for future research might include empirical studies of all stakeholders to observe whether there is any similarity between online and offline ratings of reputation. Another research opportunity would be to analyze these companies directly to identify their strategies in managing and positively influencing their online reputation. Such research could ultimately detect the most successful strategies in this regard. Finally, research should seek to corroborate the existence of the positive relationship between online reputation and financial performance suggested by the results of other studies.

## Data Availability

The datasets generated during and/or analysed during the current study are available from the corresponding author on reasonable request.

## Footnotes

1 More information available at: https://www.mapa.gob.es/es/agricultura/temas/producciones-agricolas/aceite-oliva-y-aceituna-mesa/aceite.aspx..

2 More information available at: https://www.aica.gob.es/.

3 More information available at: https://www.aica.gob.es/.
